# Circulating MicroRNAs Implicate Multiple Atherogenic Abnormalities in the Long-Term Cardiovascular Sequelae of Preeclampsia

**DOI:** 10.1093/ajh/hpy069

**Published:** 2018-05-23

**Authors:** Natalie Dayan, Kenny Schlosser, Duncan J Stewart, Christian Delles, Amanpreet Kaur, Louise Pilote

**Affiliations:** 1 Department of Medicine, Division of General Internal Medicine, McGill University Health Centre, Montreal, Quebec, Canada; 2 Research Institute, McGill University Health Centre, Montreal, Quebec, Canada; 3 Sinclair Centre for Regenerative Medicine, Ottawa Hospital Research Institute, Ottawa, Ontario, Canada; 4 Department of Cellular and Molecular Medicine, University of Ottawa, Ottawa, Ontario, Canada; 5 Institute of Cardiovascular and Medical Sciences, University of Glasgow, Scotland, UK

**Keywords:** acute coronary syndrome, blood pressure, cardiovascular disease, hypertension, microRNA, preeclampsia

## Abstract

**BACKGROUND:**

Women who have had preeclampsia (PE) are at increased risk for premature cardiovascular disease (CVD). The underlying pathophysiology of this risk remains unclear, but potentially involves subclinical vascular damage or dysfunction. Alterations in the levels of circulating microRNAs may be implicated, as they are known to play pervasive roles in vascular biology. We investigated whether levels of circulating microRNAs are altered between women with premature acute coronary syndrome (ACS), with and without a history of PE.

**METHODS:**

Women with premature ACS (age ≤ 55 years) were categorized based on a prior history of PE or normotensive pregnancy. Relative plasma levels of 372 microRNAs were initially assessed by polymerase chain reaction array in a subset of subjects (*n* = 12–13/group) matched for age, chronic hypertension, dyslipidemia, and smoking status. Candidate microRNAs were then validated in a larger cohort of ACS patients (*n* = 176).

**RESULTS:**

MicroRNAs previously linked to angiogenesis (miR-126-3p), inflammation (miR-146a-5p), and cholesterol metabolism (miR-122-5p) were significantly decreased in women with prior PE compared to women with prior normotensive pregnancy (*P* = 0.002, 0.017, and 0.009, respectively), even after adjustment for chronic hypertension.

**CONCLUSIONS:**

Circulating levels of miR-126-3p, -146a-5p, and -122-5p were significantly decreased in women with premature ACS who reported prior PE compared to those with prior normotensive pregnancy. These data provide novel insight into potential pathways that may contribute to the increased risk of CVD following PE.

Preeclampsia (PE) is a multisystem disorder of pregnancy characterized by high blood pressure and either proteinuria or other adverse conditions, and is associated with widespread maternal endothelial dysfunction.^1^ It complicates 2–8% of all pregnancies globally, and therefore imposes a significant burden on the cardiovascular health of over 300 million women worldwide.^2^ Although PE typically resolves after delivery of the placenta, exposed women have a 2-fold higher risk of premature cardiovascular disease (CVD) and cardiovascular mortality later in life, with onset 10–15 years after delivery.^2,3^ However, the molecular determinants underlying this elevated risk remain poorly defined. Increased rates of hypertension and metabolic syndrome have been noted after deliveries complicated by PE.^4^ Furthermore, markers of subclinical atherosclerosis including pulse wave velocity, augmentation index, and carotid intima-media thickness have been demonstrated to be abnormal in women with past PE compared to women with past normotensive pregnancy.^5^ However, assessment of protein biomarkers involved in endothelial cell function and angiogenic pathways linking PE with future CVD have not revealed similar insights.^5,6^ Moreover, to our knowledge no studies have assessed markers of vascular impairment after PE in the context of an acute ischemic event.

MicroRNAs (miRNAs) are small (~22 nucleotide) noncoding RNAs that regulate gene expression at the post-transcriptional level, and have been shown to play important and pervasive roles in vascular biology.^7,8^ Perturbations in the levels of circulating extracellular miRNAs may potentially reflect disease-specific mechanisms of tissue injury or transcriptional reprogramming in dysfunctional tissues, or contribute to intercellular communication.^7^ While previous studies have demonstrated the importance of miRNAs in cardiovascular health and disease,^9^ and acutely at the time of PE,^10^ their potential contribution to disease activity linking PE and future CVD has not yet been investigated. Thus, we sought to determine whether a history of PE was associated with alterations in circulating miRNA levels in plasma sampled at the time of an acute ischemic event.

## METHODS

We investigated a subset of women with premature acute coronary syndrome (ACS) identified from the GENESIS-PRAXY study,^11^ a multicenter cohort of adults aged ≤ 55 years who were hospitalized with a diagnosis of ACS between January 2009 and April 2013 from 24 centers across Canada, 1 in the United States and 1 in Switzerland. For the present study, we included all parous women with ACS (*n* = 30 with prior PE, and *n* = 146 with prior normotensive pregnancy) with adequate plasma samples drawn at study entry (Table 1). All participating sites received ethics approval from their respective ethics review boards, and participants provided informed written consent. The diagnosis of ACS was standard, based on the presence of characteristic symptoms plus at least one of: (i) significant electrocardiogram changes in ≥ 2 contiguous leads: transient ST-segment elevations of ≥ 1 mm, ST-segment depressions of ≥ 1 mm, new T-wave inversions of ≥ 1 mm, pseudonormalization of previously inverted T waves, new Q waves (one-third the height of the R wave or ≥ 0.04 seconds), new R > S wave in lead V1 (posterior myocardial infarction), new left bundle branch block; (ii) increase in cardiac enzymes levels: troponin I or T, creatine kinase-MB value > 2 × upper limit of the hospital’s normal range or if not available, then total creatine phosphokinase value > 2 × upper limit of the hospital’s normal range. Pregnancy data was collected by detailed self-reported questionnaires at study entry. Women were classified as prior PE if they reported either PE or high blood pressure in addition to proteinuria. Women who were unsure about past pregnancy complications were excluded. The time since last pregnancy was estimated using age of youngest biological child.

**Table 1.** Baseline characteristics of participants selected for this study

Characteristics	Normotensive, *n* = 146	Preeclampsia, *n* = 30	*P* value
Demographics			
Age, years, mean (SD)	49.5 (5.0)	46.1 (6.6)	0.0016
Caucasian, *n* (%)	130 (89.0)	27 (90.0)	0.87
Clinical risk factors, *n* (%)			
Hypertension	55 (37.7)	26 (86.7)	<0.0001
Diabetes	27 (18.5)	7 (23.3)	0.54
Dyslipidemia	72 (49.3)	20 (66.7)	0.08
Obesity (BMI ≥ 30 kg/m^2^)	51 (35.7)	13 (44.8)	0.35
History of CAD	31 (21.2)	11 (36.7)	0.07
Current smoking	66 (45.2)	11 (36.7)	0.39
Pregnancy characteristics			
History of fetal loss, *n* (%)	17 (11.6)	7 (23.3)	0.09
Years since pregnancy, mean (SD)	20.2 (7.5)	14.2 (8.4)	0.0003
Gestational diabetes, *n* (%)	11 (7.5)	7 (23.3)	0.009
Type of ACS, *n* (%)			
STEMI	68 (46.6)	19 (63.3)	0.09
Non-STEMI	61 (41.8)	11 (36.7)	0.60
Unstable angina	17 (11.6)	0 (0.0)	0.05
Biomarkers, mean (SD)			
Total cholesterol (mmol/l)	4.6 (1.3)	4.4 (1.5)	0.62
Low density lipoprotein (mmol/l)	2.7 (1.2)	2.5 (1.4)	0.54
Standardized troponin (μg/l)	10.2 (20.2)	20.8 (50.8)	0.07
Sflt-1 (pg/ml)	140.1 (200.0)	187.2 (265.2)	0.27
PlGF (pg/ml)	31.5 (24.8)	27.1 (14.1)	0.35
Soluble endoglin (pg/ml)	771.4 (311.1)	758.5 (244.0)	0.83

Abbreviations: ACS, acute coronary syndrome; BMI, body mass index; CAD, coronary artery disease; PlGF, placental growth factor; STEMI, ST-elevation myocardial infarction.

Venipuncture was performed on all consenting participants within 24 hours of hospital admission for ACS. Archived plasma samples (stored at −80 °C) were previously prepared by centrifuging whole blood at 3,000 rpm for 10 minutes at 4 °C. Poor quality specimens with evidence of hemolysis or possible RNA degradation were not included among the final cohort of 176 subjects. A 2-phase discovery and validation approach was used to identify candidate plasma miRNAs that were associated with prior PE at the time of ACS. Circulating extracellular miRNAs were measured according to previously published methods.^12^ Briefly, total RNA was extracted from 200 µl of EDTA plasma using the miRNeasy mini kit (Qiagen). A fixed volume of RNA eluate was converted to cDNA with the miScript II RT kit (Qiagen), and quantified on a Biorad CFX384 PCR machine using miScript primers and polymerase chain reaction reagents (Qiagen). MiRNA levels were calculated as relative expression units, 2^-ΔCq^, normalized to a cel-miR-39 spike-in control and log_e_ transformed for statistical analysis.

The derivation cohort used for miRNA screening included 12–13 ACS patients each with a history of PE or normotensive pregnancy, matched on age, hypertension, smoking status, and dyslipidemia. No miRNAs passed a false discovery rate correction for multiple comparisons at *P* < 0.05. Therefore, miRNA candidates were initially prioritized according to unadjusted *P*-values, and then a subset were selected for further validation in the entire patient cohort (*n* = 176) based on the magnitude of difference between exposure groups and mean circulating level.

Baseline characteristics and circulating miRNA values were compared in women with and without a history of PE using *t*-test, chi-square, or Mann–Whitney rank sum tests as appropriate. Multivariate linear and logistic regression were performed to assess the association between candidate miRNAs modeled as dependent variables (continuous or dichotomized at mean circulating levels) with a history of PE or normotensive pregnancy adjusted for current hypertension, with statistical significance at 2-sided alpha 0.05. Spearman correlation coefficients (ρ) were determined between circulating levels of target miRNAs and several previously reported biological markers,^6^ including total cholesterol (mg/dl), low-density lipoprotein (mg/dl), high-denisity lipoprotein (mg/dl), standardized troponin, soluble fms-like tyrosine kinase (sflt-1, pg/ml), Placental Growth Factor (PlGF, pg/ml), and soluble endoglin (pg/ml).

## RESULTS

Women with prior PE were significantly younger at the time of ACS compared to women with prior normotensive pregnancy (46.1 ± 6.6 years vs. 49.5 ± 5.0 years, *P* = 0.002), and accordingly had been pregnant more recently (14.2 years vs. 20.2 years prior, *P* = 0.0005). Women with prior PE were also more likely to have a diagnosis of chronic hypertension (86.7% with prior PE vs. 37.7% with prior normotensive pregnancy, *P* < 0.0001); however, mean systolic blood pressure at ACS presentation did not significantly differ between groups.

We screened 372 circulating miRNAs in the derivation cohort. A total of 16 miRNA candidates were identified, which showed differential levels (based on an unadjusted *P* < 0.05 cutoff) between women with prior PE compared to those with prior normotensive pregnancy, including 10 miRNAs that were increased (1.3–2.0-fold change) and 6 miRNAs that were decreased (1.3–2.8-fold change) in PE. Of these 16 miRNAs, we further prioritized 3 candidates (miR-122-5p, miR-146a-5p, and miR-126a-3p) that met predefined criteria for assessment in the larger validation cohort. These miRNAs have potentially relevant biological functions, including hepatic lipid metabolism (miR-122-5p), angiogenesis (miR-126-3p), and anti-inflammatory (miR-146a-5p), and all have been implicated in the atherogenic process.^13^

**Figure 1.**
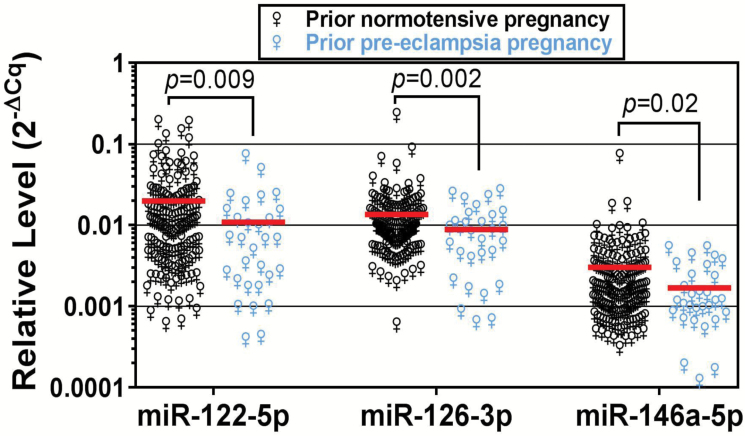
Plasma levels of miR-122-5p, miR-126-3p, and miR-146a-5p in a cohort of women with premature ACS, stratified according to a history of preeclampsia (*n* = 30) or normotensive pregnancy (*n* = 146).

In the larger cohort (*n* = 176; *n* = 30 with prior PE) all 3 miRNAs were significantly (*P* < 0.05) downregulated in plasma of ACS patients with a history of prior PE vs. those with prior normotensive pregnancy (Figure 1). After adjustment for chronic hypertension, miRNA-122-5p remained significantly inversely associated with a history of PE (odds ratio [OR] 0.66, 95% confidence interval [CI] 0.47–0.92), as were miR-126-3p (OR 0.48, 95% CI 0.29–0.78) and miR-146a-5p (OR 0.57, 95% CI 0.35–0.91) when each of these miRNAs were dichotomized at their mean circulating level.

In a correlation analysis of log-transformed miRNAs and several other relevant biomarkers, miR-122-5p was negatively correlated with total cholesterol (ρ = −0.21, *P* = 0.03) and standardized troponin (ρ = −0.25, *P* = 0.007). miR-126-3p was negatively correlated with standardized troponin (ρ = −0.19, *P* = 0.04), and positively correlated with Placental Growth Factor (ρ = 0.14, *P* = 0.05). miR-146a-5p was positively correlated with endoglin (ρ = 0.21, *P* = 0.006) and Placental Growth Factor (ρ = 0.25; *P* = 0.0007).

## DISCUSSION

The aim of this present study was to explore whether miRNA patterns would distinguish women with prior PE from women with prior normotensive pregnancy at the time of ACS. We hypothesized that significant differences in circulating miRNAs might discern potential cellular mechanisms of premature ischemic heart disease in 2 groups of women based on their prior pregnancy history.

Our findings suggest that PE may promote subclinical perturbations in multiple atherogenic pathways that persist even after resolution of PE, which could predispose affected women to future CVD. A recent review on miRNAs implicated in the pathogenesis of atherosclerosis identified these same candidate miRNAs as playing central roles.^13^ miR-126 is highly expressed in endothelial cells^14^ and inhibits the expression of vascular cell adhesion molecule 1 (VCAM1). miR-126 also regulates endothelial proliferation after vascular injury. Thus, down-regulation of miR-126 is associated with reduced endothelial proliferation, and promotes atherogenesis.^13,14^ MiR-146 has an important role in suppressing endothelial activation and inflammatory pathway regulation by potentiating T-cell receptors.^15^ Decreased levels of miR-146 can lead to unsuppressed inflammation and endothelial activation. miR-122 regulates fat and cholesterol metabolism, and genetic deletion of miR-122 in mice has been shown to increase lipid synthesis, inflammation, and fibrosis in the liver.^13,16^ Thus, alterations in these miRNAs may potentiate various steps of the atherogenic process in women who have had PE. We also found correlations between circulating levels of miRNAs and other biomarkers of angiogenesis, cardiac damage and lipid metabolism that may provide insights into the biological action of these miRNAs, but due to the complexity of miRNA effects on gene expression such exploratory data should be interpreted with caution. The potential interplay between perturbations in the circulating extracellular levels of these miRNAs and their epigenetic intracellular roles in women with a history of PE deserves further study.

Our data should be considered hypothesis-generating, since the observational nature of this study with retrospective ascertainment of pregnancy history precludes discussion of any causal link between these altered circulating miRNAs, PE and ACS. Furthermore, the lack of a healthy comparison group limits our understanding of the impact of the ACS itself on circulating extracellular miRNAs. Future studies that compare miRNA profiles in healthy women with women who have ischemic heart disease and correlate with past pregnancy events will help to clarify this association. Nevertheless, this is the first study to implicate a potential role for dysregulated circulating miRNAs in the long-term cardiovascular sequelae of PE. We identify novel molecular targets that merit further investigation in independent patient cohorts, and interrogation in animal models, to help delineate whether these altered miRNAs are markers or mediators of underlying disease activity.

## DISCLOSURE

The authors declared no conflict of interest.
